# Model based on clinical characteristics to identify patients with neurogenic oropharyngeal dysphagia

**DOI:** 10.15649/cuidarte.3861

**Published:** 2024-09-01

**Authors:** Juan Camilo Suárez-Escudero, Sara González-Franco, Isabela Franco-Sánchez, Elizabeth Gómez-Ríos, Lillyana Martínez-Moreno

**Affiliations:** 1 Universidad Pontificia Bolivariana. Medellín, Colombia. Universidad CES. Medellín, Colombia. E-mail: juanca.suarez@upb.edu.co Universidad Pontificia Bolivariana Universidad Pontificia Bolivariana Medellín Colombia juanca.suarez@upb.edu.co; 2 Universidad Pontificia Bolivariana. Medellín, Colombia. E-mail: sara.gonzalezf@upb.edu.co Universidad Pontificia Bolivariana Universidad Pontificia Bolivariana Medellín Colombia sara.gonzalezf@upb.edu.co; 3 Universidad Pontificia Bolivariana. Medellín, Colombia. E-mail: isabela.francos@upb.edu.co Universidad Pontificia Bolivariana Universidad Pontificia Bolivariana Medellín Colombia isabela.francos@upb.edu.co; 4 Universidad Pontificia Bolivariana. Medellín, Colombia. E-mail: eligomez.qmezros@qmail.com Universidad Pontificia Bolivariana Universidad Pontificia Bolivariana Medellín Colombia eligomez.qmezros@qmail.com; 5 Organización Fonoaudiológica OFA IPS. Medellin, Colombia. E-mail: liliana.m@ofa.com.co Organización Fonoaudiológica OFA IPS Medellin Colombia liliana.m@ofa.com.co

**Keywords:** Deglutition, Deglutition Disorders, Central Nervous System Diseases, Neuromuscular Diseases, Signs and Symptoms, Case-Control Studies, Deglución, Trastornos de Deglución, Enfermedades del Sistema Nervioso Central, Enfermedades Neuromusculares, Signos y Síntomas, Estudios de Casos y Controles, Deglutição, Transtornos de Deglutição, Doenças do Sistema Nervoso Central, Doenças Neuromusculares, Sinais e Sintomas, Estudos de Casos e Controles

## Abstract

**Introduction:**

Neurogenic oropharyngeal dysphagia is a form of functional dysphagia usually caused by neurological and neuromuscular diseases, which produces several secondary complications. To improve its detection and characterization, models are emerging that integrate clinical variables to complement the physical examination of swallowing.

**Objective:**

Develop an explanatory model to differentiate patients with neurogenic oropharyngeal dysphagia.

**Materials and Methods:**

Case control study based on a set ofdata derived from the clinical examination of swallowing with neurological emphasis carried out in a sample of patients with neurogenic oropharyngeal dysphagia of neurological and neuromuscular causes (cases), and in healthy people (controls).

**Results:**

158 clinical variables were compared between both groups, where those with the greatest classification capacity were identified, integrated into an explanatory binary logistic regression model made up of nine variables: two history, two symptoms, three physical examination signs and two signs after consistency/volume test with food. The dependent variable was the category of being healthy or patient and the covariates were the clinical variables. Parameters reached by the model: Akaike information criterion 102 and Nagelkerke R2 0.78.

**Discussion:**

The nine variables that entered the model, together, largely explain the presence of neurogenic oropharyngeal dysphagia, and are accessible by physical examination of swallowing.

**Conclusions:**

The model obtained can improve and/or complement the evaluation process carried out in patients with dysphagia of functional causes, neurological and neuromuscular diseases, in screening and diagnostic characterization processes.

## Resumen.

**Introducción:**

La disfagia orofaríngea neurogénica es una forma de disfagia funcional causada habitualmente por enfermedades neurológicas y neuromusculares, que produce varias complicaciones secundarias. En pro de mejorar su detección y caracterización surgen modelos que integren variables clínicas para complementar el examen físico de la deglución.

**Objetivo:**

Desarrollar un modelo explicativo para diferenciar pacientes con disfagia orofaríngea neurogénica.

**Materiales y Métodos:**

Estudio de casos y controles basado en un conjunto de datos derivados del examen clínico de la deglución con énfasis neurológico realizado en una muestra de pacientes con disfagia orofaríngea neurogénica de causas neurológicas y neuromusculares (casos), y en personas sanas (controles).

**Resultados:**

Se compararon 158 variables clínicas entre ambos grupos, donde se identificaron aquellas con mayor capacidad de clasificación, integradas en un modelo explicativo de regresión logística binaria conformada por nueve variables: dos antecedentes, dos síntomas, tres signos de examen físico y dos signos tras prueba de consistencia/volumen con alimentos. La variable dependiente fue la categoría de ser sano o paciente y las covariables fueron las variables clínicas. Parámetros que alcanzó el modelo: criterio de información de Akaike 102 y R2 de Nagelkerke 0,78.

**Discusión:**

Las nueve variables que ingresaron al modelo, en conjunto, logran explicar en gran medida la presencia de disfagia orofaríngea neurogénica, y son accesibles por examen físico de la deglución.

**Conclusiones:**

El modelo obtenido puede mejorar y/o complementar el proceso evaluativo que se realiza en pacientes con disfagia de causas funcionales, enfermedades neurológicas y neuromusculares, en procesos de tamizaje y caracterización diagnóstica.

## Resumo.

**Introdução:**

A disfagia orofaríngea neurogênica é uma forma de disfagia funcional geralmente causada por doenças neurológicas e neuromusculares, que produz diversas complicações secundárias. Com o objetivo de melhorar a sua detecção e caracterização, estão surgindo modelos que integram variáveis clínicas para complementar o exame físico da deglutição.

**Objetivo:**

Desenvolver um modelo explicativo para diferenciar pacientes com disfagia orofaríngea neurogênica.

**Materiais e Métodos:**

Estudo caso-controle baseado em um conjunto de dados derivados do exame clínico da deglutição com ênfase neurológica realizado em uma amostra de pacientes com disfagia orofaríngea neurogênica de causas neurológicas e neuromusculares (casos) e em pessoas saudáveis (controles).

**Resultados:**

Foram comparadas 158 variáveis clínicas entre os dois grupos, onde foram identificadas aquelas com maior capacidade de classificação, integradas em um modelo explicativo de regressão logística binária composto por nove variáveis: dois antecedentes, dois sintomas, três sinais de exame físico e dois sinais após consistência /volume teste com alimentos. A variável dependente foi a categoria de ser saudável ou paciente e as covariáveis foram as variáveis clínicas. Parâmetros alcançados pelo modelo: critério de informação de Akaike 102 e Nagelkerke R2 0,78.

**Discussão:**

As nove variáveis que entraram no modelo, juntas, explicam em grande parte a presença de disfagia orofaríngea neurogênica, e são acessíveis pelo exame físico da deglutição.

**Conclusões:**

O modelo obtido pode melhorar e/ou complementar o processo de avaliação realizado em pacientes com disfagia de causas funcionais, doenças neurológicas e neuromusculares, em processos de triagem e caracterização diagnóstica.

## Introduction

Dysphagia is an alteration of the transit of food and liquids from the oral cavity to the hypopharynx and esophagus. The great variety of structures involved in the swallowing process suggests that different pathophysiological mechanisms may result in dysphagia, depending on the underlying disease, the associated structural and functional impairment^1^.

Its prevalence in the general population is between 8.4% and 16%^2^. It is more prevalent in older adults where the aging process increases the probability of acquiring neurological, respiratory, cardiovascular and gastrointestinal comorbidities, and these in turn can generate dysphagia^3^.

Dysphagia as a symptom is a sensation ofstickiness and obstruction ofthe passage offood through the mouth, pharynx or esophagus, secondary to a difficulty in moving the bolus safely from the oral cavity to the stomach, without aspiration^4^^, ^^5^. But dysphagia is not always obvious or symptomatic, and can present subtly as weight loss, a notable increase in the time needed to eat, lateral or tilting movements of the head when eating, repeated need to drink water with food, even presence of repeated respiratory infections^6^^, ^^7^. In certain cases it is only one of several manifestations of a systemic disease, but from a clinical and epidemiological perspective it is more likely that it has a neurological etiology^8^^, ^^9^. By anatomical and clinical location, it is classified into oropharyngeal (OD) and esophageal dysphagia, and by its etiology into structural (mechanical), motor (propulsion) and functional causes^10^^, ^^11^.

OD, also known as transfer dysphagia, is characterized by difficulty in initiating swallowing, plus problems moving the bolus due to compromise of the oral, oral preparatory or pharyngeal phase of swallowing^11^. Apart from being a clinical form of dysphagia, it is a disorder accompanying several diseases, especially neurological and neuromuscular, both in children and adults. A key aspect is that it is heterogeneous and complex^1^ and is frequently associated with secondary pulmonary and nutritional complications^12^^, ^^13^.

Functional causes are characterized by deteriorating swallowing physiology^11^. Most functional causes of OD are related to alterations in the central neurological control ofthe oral and pharyngeal phase of swallowing, the swallowing reflex, the modulation of peristalsis or neuromuscular coordination of the upper esophageal sphincter, or in the action and synchronization of the muscle effectors involved in it^14^. In other words, there is a disorder in oropharyngeal functioning, which leads to three major alterations: a) poor bolus propulsion; b) poor oropharyngeal reconfiguration during swallowing; and c) poor opening of the upper esophageal sphincter^15^. Neurological disorders explain 70% to 80% of the etiology of OD^16^. From the above, the category and term of neurogenic oropharyngeal dysphagia (NOD) arises, usually caused by stroke, amyotrophic lateral sclerosis (ALS), Parkinson's disease (PD) and myasthenia gravis (MG)^3^.

In patients with neurological and neurodegenerative entities, the frequency of NOD is reported between 30% to 82%^15^^, ^^17^, and as a geriatric syndrome it affects 56% to 78% of institutionalized older adults and 44% of older adults admitted to a hospital. general hospital18. Between 400,000 to 800,000 people in the world develop NOD per year^19^.

Dysphagia in general produces a wide spectrum of symptoms and signs that are used in screening methods, in formal clinical evaluations and in instrumental tests, to detect its presence and severity. The standard formal clinical evaluation is the bedside clinical examination of swallowing (CES). *Swallow examination*), which consists of a process based on clinical history and execution of an exhaustive physical examination of the oral, pharyngeal, and laryngeal anatomy plus neurological aspects focused on sensory, motor, cognitive, behavioral, and language function^20^^, ^^21^; Some authors define it as a clinical checklist carried out mainly by speech therapists trained in swallowing^20^.

Predictive models have been published in swallowing applied in acute dysphagia after radiotherapy in patients with head and neck cancer^22^, persistent dysphagia in stroke^23^, risk of aspiration^24^ and post-stroke dysphagia recovery^25^. However, there is a lack of logistic regression models and flow charts based on the joint behavior of symptoms and signs to detect or classify patients with NOD, which could be a complement to CES and reference tests such as video fluoroscopy of swallowing (VFSS) and functional endoscopic evaluation of swallowing (FEES).

The objective of this study was to develop an explanatory model (algorithm) based on clinical characteristics of the CES and gold motor test with different consistencies/volume, to differentiate healthy people from patients with NOD of neurological and neuromuscular causes.

## Materials and Methods

Case-control study based on a set of data that are stored in the dataset entitled MedSwallowDB : clinical database derived from the CES with neurological emphasis carried out in patients with NOD of neurological and neuromuscular causes (cases), and in healthy people (controls)^26^.

A sample size was previously reported to identify the clinical variables that, together with the integration of non-invasive signals (surface electromyography and laryngeal accelerometry), would improve sensitivity in the differentiation of patients with NOD of neurological and neuromuscular causes from healthy patients^27^. Briefly, an CES sensitivity of 80%^7^, a 15% increase in sensitivity, a power of 80%, and a confidence of 95% were assumed. It was possible to recruit 210 people (107 patients and 103 healthy), to select (according to criteria) 166 people (86 cases and 80 controls), who were all analyzed to answer the question: What clinical characteristics of the CES and gold test motor with different consistencies/volume, when integrated into a model, can they differentiate healthy patients from patients with NOD due to neurological and neuromuscular causes?

Case eligibility criteria: age >18 years, both sexes; presence of NOD of at least one month or more of evolution. Diagnosis of central neurological or neuromuscular pathologies that in their evolution have caused DO. Total score on the Eating instrument Assessment Tool (EAT-10) >3 points. Symptoms of cough, food stuck in the throat or sensation of choking in relation to swallowing food or changes in voice when swallowing, difficulties in initiating swallowing or need for multiple swallows to swallow food visualized on physical examination. Patients with esophageal dysphagia, mechanical, propulsive or iatrogenic dysphagia were excluded; irradiated facial and/or cervical region skin; orofacial or cervical edema or hematomas; recent (<3 months) surgical dissection of neck skin; severe hypoxemia (ambient oxygen saturation <80%, unresponsive to oxygen therapy); late-stage dementia that prevents understanding of simple commands to chew and swallow; present congenital structural malformations in the oral cavity, tongue or neck; have a diagnosis of Sjogren's disease and be undergoing active endodontic procedures.

Eligibility criteria for controls: age >18 years, both sexes; without diagnosis of dysphagia, central, peripheral or neuromuscular neurological pathologies. Total score on EAT-10 <3 points. Absence of comorbidities such as head and neck cancer, chronic obstructive pulmonary disease (COPD), and surgical procedures in the lower 2/3 parts of the face or neck, or use of botulinum toxin in the head/ neck. Healthy people in active endodontic procedures were excluded; with the presence of congenital malformations in the oral cavity, tongue and neck; diagnosis of Sjogren's disease and cognitive impairment.

The patients were recruited in twelve private offices of professionals in speech therapy and swallowing, ten health care institutions with dysphagia services, four health care institutions for older adults and three patient-founded organizations located in the Aburrá and San Nicolás valleys in the department of Antioquia, Colombia.

Healthy people were recruited in two socialization and leisure centers for older adults, two universities, a community action board located in the Aburrá valley (Medellín) and from healthy relatives of the patients.

The validation of the eligibility criteria for the selection of the case group was carried out by a neurologist with experience in NOD care, with the support of a speech pathologist trained in swallowing/dysphagia. The validation of criteria for the selection of controls was carried out by a specialist doctor trained in swallowing/dysphagia. The recruitment, selection and obtaining of variables in both groups were carried out between the first semester of 2019 and the second semester of 2022, so that the selection of the controls was simultaneous with the recruitment and selection of the cases.

In both groups, the EAT-10 instrument was completed with validation for Colombia2 , the same protocol was applied to perform the CES with the purpose of obtaining comparable data, and the groups (cases/controls) were matched by age.

The CES included anamnesis focused on medical history, swallowing characteristics and symptoms of dysphagia, plus evaluation of the anatomy, function, sensitivity and reflexes of the swallowing system (focused on the oral and pharyngeal phase) through physical examination of the oral cavity, respiratory system, of lower cranial nerves plus olfactory, trigeminal and facial nerves, execution of orofacial praxis and lung auscultation. We asked about consumption of medications that can modify swallowing (E.g. neuroleptics, barbiturates, anxiolytics, non-steroidal anti-inflammatory drugs, muscle relaxants, anticholinergics and tricyclic antidepressants). Measurement of height/weight and oxygen saturation was included, and gold motor testing was performed using therapeutic foods with different consistencies/volumes (orally).

The gold motor test included swallowing 5, 10, and 20 ml of thick yogurt without fruit pieces, three grams of dry, salty cracker, and 5, 10, and 20 ml of water. Between each consistency, the person was asked to take a swallow of saliva. In both groups, the preparation of the bolus, attachment, lip competence, ability to manage secretions, presence or not of fatigue, throat clearing, oral waste and cough, laryngeal elevation, need or not for multiple swallows, presence of nasal regurgitation and qualification of the swallowing reflex.

The variables obtained were grouped into history, swallowing characteristics, symptoms of dysphagia, signs on physical examination and clinical findings on the gold motor test. The final EAT-10 score was considered as a quantitative variable in the symptom group. The quantitative variables of oxygen saturation, weight, height and body mass index (BMI) are part of the set of signs on the physical examination.

Descriptive statistics were used on the set of variables previously evaluating normality using the Shapiro-Wilk test, and cross-sectional analysis to compare the set of variables and identify those with statistically significant differences, by performing comparisons. and construction of contingency tables between cases/controls, and thus obtain exploratory odds ratios (OR) accompanied by 95% confidence intervals. The significance test (p value) in the qualitative variables was obtained using Chi square or Fisher's exact test, identifying those variables with statistically very significant differences (p value <0.005) as a variable reduction method.

Significance testing was performed on the quantitative variables using the Mann-Whitney U (in variables with non-normal distribution) and Student 's T or Welch's T (in variables with normal distribution and Levene's homogeneity test). Then those variables with a p value <0.005 were identified.

Once the clinical variables with very significant statistical differences were identified, several binary logistic regression (BLR) models were constructed, explanatory and non-predictive models.

Parameters for the construction of the BLR models: a) the dependent variable was the category of being healthy or patient (reference level: healthy); b) the covariates were the previously identified clinical variables; c) evaluation of collinearity using the variance inflation factor (VIF) with ideal values between 1 and 3; d) Akaike information criterion (AIC) as a measure of model fit; e) explanation of the model through R2 of Nagelkerke (R2N); f) OR and 95% confidence intervals; and g) the assumption of independence of observations was guaranteed by the study design, where the controls came from different places than the cases.

All processing was performed in the free statistical program Jamovi version 2.2.5.0. Informed consent is obtained from all the people studied. Study approved by the Health Research Ethics Committee of the Universidad Pontificia Bolivariana (minutes No. 7, June 1, 2017), research ethics committee Fundación Hospitalaria San Vicente Paúl (minutes No. 35-2018, December 21, 2018) and research ethics committee of the Somer Clinic (minutes N°01-2019, February 8, 2019).

## Results

**Figure 1:**
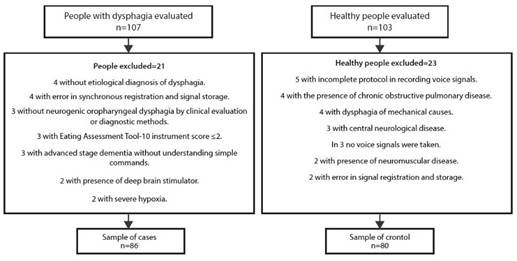
Flowchart of the recruitment and selection process for cases and controls.

Between March 2019 and December 2021, 210 people were evaluated, 107 (51%) patients with OD and 103 (49%) healthy. Final sample of 166 people: 86 cases with NOD due to neurological and neuromuscular causes, and 80 controls (see Figure 1). Both groups with the same evaluation protocol.

The male sex was more frequent in the cases with 59.30% (51/86) and in controls it was the female sex with 53.75% (43/80), without statistically significant differences (p=0.092). The median age in both groups was close to 61 years (interquartile range: 51:67), without statistically significant differences (p=0.798). Both groups similar in terms of sex and age. Table 1 details the sociodemographic and background characteristics in both groups.

**Table 1:** Sociodemographic characteristics and background of the study population.

Variables	Cases (86)	Controls (80)
Age (years). Median (IQR)	60.5 (48.3:68)	61.5 (54:66.3)
Sex %(n)		
Female	40.70 (35)	53.75 (43)
Male	59.30 (51)	46.25 (37)
Comorbidities %(n)		
Cardiovascular	40.70 (35)	30.00 (24)
Thyroid	10.47 (9)	13.75 (11)
Dyslipidemia	10.47 (9)	13.75 (11)
Gastrointestinal	9.30 (8)	12.50 (10)
Rheumatological	10.47 (9)	10.00 (8)
Diabetic	12.79 (11)	6.25 (5)
Neurological	19.77 (17)	3.75 (3)
Respiratory	18.60 (16)	3.75 (3)
Emotional	6.98 (6)	3.75 (3)
Presence of deficiencies %(n)		
Multiple	43.02 (37)	0 (0)
Physical	39.53 (34)	0 (0)
Intellectual	4.65 (4)	0 (0)
None	12.79 (11)	100 (80)
Reception of therapies %(n)		
Physical therapy	38.37 (33)	0 (0)
Swallowing therapy	34.88 (30)	0 (0)
Respiratory therapy	5.81 (5)	0 (0)
Respiratory history %(n)		
Intubation longer than one week	13.95 (12)	2.50 (2)
Tracheostomy greater than six months	8.14 (7)	0 (0)
Recurrent pneumonia	4.65 (4)	0 (0)
Aspiration pneumonia	12.79 (11)	0 (0)

*IQR: inter-quartile range.*

In the cases, the etiology of NOD was due to central neurological causes in 88.37% (76/86) and due to neuromuscular causes in 11.62% (10/86), and the median time of evolution of the NOD was 1.3 years (interquartile range: 0.6:3).

In both groups, a set of 158 clinical variables was obtained, grouped into 25 history, 38 swallowing characteristics and symptoms of dysphagia, 48 signs on physical examination, and 47 clinical findings from the gold motor test with consistencies/volumes.

Of the set of variables obtained from the CES, 64.55% (102/158) had statistically significant differences (p<0.05) between cases and controls, of which 80.39% (82/102) presented statistically very significant differences. significant (p<0.005) and formed the set of clinical variables to be entered for the construction of the BLR model. Table 2 details the 82 qualitative variables, grouped by history, swallowing characteristics, symptoms, signs and findings on the gold motor test, with very significant statistical differences accompanied by p and OR value.

**Table 2:** Qualitative variables of the clinical swallowing examination with very significant statistical differences (p<0.005) between cases and controls.

Variables	Cases %(n) (86)	Controls %(n) (80)	p value	OR (95% CI)
Background				
Neurological comorbidity §	19.77 (17)	3.75 (3)	0.002	6.32 (1.78 - 22.50)
Respiratory comorbidity a	18.60 (16)	3.75 (3)	0.003	5.87 (1.64 - 21)
Multiple permanent deficiency	43.02 (37)	0 (0)	<0.001	122 (7.32 - 2031)
Permanent physical impairment	39.53 (34)	0 (0)	<0.001	106 (6.35 - 1763)
Take medication that can modify swallowing	30.23 (26)	7.50 (6)	<0.001	5.34 (2.07 - 13.80)
Intubation longer than one week	13.95 (12)	2.50 (2)	0.008	6.32 (1.37 - 29.20)
Aspiration pneumonia	12.79 (11)	0 (0)	<0.001	24.5 (1.42 - 423)
Receiving physical therapy	38.37 (33)	0 (0)	<0.001	101 (6.05 - 1681)
Receiving swallowing therapy	34.88 (30)	0 (0)	<0.001	86.90 (5.21 - 1451)
Swallowing characteristics				
No oral tolerance to clear liquids	29.07 (25)	0 (0)	<0.001	66.80 (3.99 - 1118)
No oral tolerance to wet solids	17.44 (15)	1.25 (1)	<0.001	16.70 (2.15 -130)
No oral tolerance to dry solids	46.51 (40)	0 (0)	<0.001	140 (8.42 - 2334)
No oral tolerance to hard solids	69.77 (60)	0 (0)	<0.001	368 (22 - 6152)
No oral tolerance to mixed consistencies	31.40 (27)	0 (0)	<0.001	74.40 (4.45 - 1245)
Reports problems starting or starting to swallow	32.56 (28)	0 (0)	<0.001	78.40 (4.69 - 1311)
Feeling of food stuck after swallowing	89.53 (77)	1.25 (1)	<0.001	676 (83.6 - 5463)
Symptoms				
Cough before swallowing	17.44 (15)	2.50 (2)	0.002	8.24 (1.82 - 37.30)
Cough after swallowing	73.26 (63)	0 (0)	<0.001	435 (25.90 - 7301)
Choking sensation after swallowing	46.51 (40)	2.50 (2)	<0.001	33.90 (7.83 - 147)
Loss of appetite	32.56 (28)	3.75 (3)	<0.001	12.40 (3.59 - 42.80)
Fear to eat	23.26 (20)	1.25 (1)	<0.001	23.90 (3.13 - 183)
Loss of taste	19.77 (17)	2.50 (2)	<0.001	9.61 (2.14 - 43.10)
Weight loss	41.86 (36)	10.00 (8)	<0.001	6.48 (2.78 - 15.10)
Continuous feeling of fullness	25.58 (22)	6.25 (5)	<0.001	5.16 (1.85 - 14.40)
Nasal regurgitation of food	12.79 (11)	0 (0)	<0.001	24.50 (1.42 - 423)
Difficulty chewing	40.70 (35)	2.50 (2)	<0.001	26.80 (6.17 - 116)
Odynophagia	13.95 (12)	0 (0)	<0.001	27 (1.57 - 464)
Pain in the chest	12.79 (11)	1.25 (1)	0.004	11.60 (1.46 - 91.90)
Signs on physical examination				
Commitment march	60.47 (52)	0 (0)	<0.001	245 (14.7 - 4083)
Sensory involvement of the mandibular nerve (V3)	18.60 (16)	0 (0)	<0.001	37.70 (2.22 - 640)
Altered jaw reflex	39.53 (34)	2.50 (2)	<0.001	25.50 (5.87 - 111)
Central facial paralysis	13.95 (12)	0 (0)	<0.001	27 (1.57 - 464)
Absent right gag reflex	20.93 (18)	51.25 (41)	<0.001	0.25 (0.12 - 0.49)
Absent left gag reflex	18.60 (16)	52.50 (42)	<0.001	0.20 (0.10 - 0.41)
Altered lingual strength	18.60 (16)	1.25 (1)	<0.001	18.10 (2.33 - 140)
Altered lingual coordination	12.79 (11)	1.25 (1)	0.004	11.60 (1.46 - 91.90)
Presence of lingual fasciculation	27.91 (24)	1.25 (1)	<0.001	30.60 (4.03 - 232)
Presence of lingual atrophy	11.63 (10)	0 (0)	0.002	22.10 (1.27 - 384)
Presence of dry mouth	22.09 (19)	0 (0)	<0.001	46.50 (2.76 - 785)
Difficulty in gesture: protruding lips	18.60 (16)	1.25 (1)	<0.001	18.10 (2.33 - 140)
Difficulty in gesture: smiling	16.28 (14)	0 (0)	<0.001	32.20 (1.89 - 549)
Difficulty in gesture: bringing lips together on the right	26.74 (23)	3.75 (3)	<0.001	9.37 (2.69 - 32.70)
Difficulty in gesture: bringing lips together on the left	27.91 (24)	3.75 (3)	<0.001	9.94 (2.86 - 34.50)
Difficulty in gesture: bringing lips together on both sides	29.07 (25)	5.00 (4)	<0.001	7.79 (2.57 - 23.60)
Clinical findings in the motor gold test with oral consistency/volume				
Cannot prepare bolus with cookie	16.28 (14)	0 (0)	<0.001	32.20 (1.89 - 549)
Cannot prepare bolus with water	12.79 (11)	0 (0)	<0.001	24.50 (1.42 - 423)
Non-attachment with saliva	20.93 (18)	2.50 (2)	<0.001	10.30 (2.31 - 46.10)
Non-attachment with cookie	33.72 (29)	7.50 (6)	<0.001	6.27 (2.44 -16.10)
Non-attachment with water	26.74 (23)	6.25 (5)	<0.001	5.48 (1.97 - 15.20)
There is no lip competition for saliva	16.28 (14)	0 (0)	<0.001	32.20 (1.89 - 549)
There is no lip competition for yogurt	20.93 (18)	0 (0)	<0.001	43.50 (2.57 - 735)
There is no lip competition for cookie	24.42 (21)	0 (0)	<0.001	52.80 (3.14 - 889)
There is no lipstick competition for water	27.91 (24)	1.25 (1)	<0.001	30.60 (4.03 - 232)
Inability to handle secretions with saliva	53.49 (46)	0 (0)	<0.001	185 (11.10 - 3077)
Inability to handle secretions with yogurt	52.33 (45)	1.25 (1)	<0.001	86.70 (11.50 - 652)
Inability to handle secretions with cookie	54.65 (47)	0 (0)	<0.001	194 (11.60 - 3223)
Inability to handle secretions with water	55.81 (48)	0 (0)	<0.001	302 (12.20 - 3377)
Presence of fatigue with saliva	10.47 (9)	0 (0)	0.003	19.70 (1.13 - 345)
Presence of fatigue with yogurt	12.79 (11)	0 (0)	<0.001	24.5 (1.42 - 423)
Presence of fatigue with cookie	10.47 (9)	0 (0)	0.003	19.70 (1.13 - 345)
Presence of fatigue with water	16.28 (14)	0 (0)	<0.001	32.20 (1.89 - 549)
Presence of throat clearing with saliva	31.40 (27)	1.25 (1)	<0.001	36.20 (4.78 - 274)
Presence of throat clearing with yogurt	43.02 (37)	1.25 (1)	<0.001	59.70 (7.93 - 449)
Presence of throat clearing with cookie	39.53 (34)	2.50 (2)	<0.001	25.50 (5.87 - 111)
Presence of throat clearing with water	48.84 (42)	1.25 (1)	<0.001	75.40 (10 - 567)
Presence of oral residue with saliva	32.56 (28)	8.75 (7)	<0.001	5.03 (2.05 - 12.3)
Presence of oral residues with yogurt	56.98 (49)	6.25 (5)	<0.001	19.90 (7.30 - 54)
Presence of oral residue with cookie	62.79 (54)	17.50 (14)	<0.001	7.96 (3.86 - 16.40)
Presence of oral residues with water	37.21 (32)	5.00 (4)	<0.001	11.30 (3.76 - 33.70)
Cough with saliva	17.44 (15)	0 (0)	<0.001	34.90 (2.05 - 594)
Yogurt cough	17.44 (15)	1.25 (1)	<0.001	16.70 (2.15 - 130)
Cough with cookie	16.28 (14)	0 (0)	<0.001	32.20 (1.89 - 549)
Cough with water	33.72 (29)	0 (0)	<0.001	82.60 (4.95 - 1380)
Reduced laryngeal elevation when swallowing saliva	58.14 (50)	0 (0)	<0.001	223 (13.40 - 3710)
Reduced laryngeal elevation when swallowing yogurt	58.14 (50)	0 (0)	<0.001	223 (13.40 - 3710)
Reduced laryngeal elevation when swallowing cookie	53.49 (46)	0 (0)	<0.001	185 (11.10 - 3077)
Reduced laryngeal elevation when swallowing water	59.30 (51)	0 (0)	<0.001	234 (14 - 3891)
Need for multiple swallows for saliva	37.21 (32)	0 (0)	<0.001	96 (5.76 - 1601)
Need for multiple swallows for yogurt	79.07 (68)	10.00 (8)	<0.001	34 (13.90 - 83.30)
Need for multiple swallows for cookie	63.95 (55)	15.00 (12)	<0.001	10.10 (4.72 - 21.40)
Need for multiple swallows for water	73.26 (63)	6.25 (5)	<0.001	41.10 (14.80 - 114)
Impaired swallowing reflex	60.47 (52)	0 (0)	<0.001	245 (14.70 - 4083)

*§ neurological comorbidity: migraine, epilepsy and/or abnormal movements.* ^*A*^ *respiratory comorbidity: chronic obstructive pulmonary disease, obstructive sleep apnea, asthma and/or rhinitis. p-value: Chi squared and Fisher's exact test. OR: Odds Ratio. CI: 95% Confidence Interval.*

Table 3 shows the quantitative variables of the CES with very significant statistical differences between both groups.

**Table 3:** Quantitative variables of the clinical swallowing examination with very significant statistical differences (p<0.005) between cases and controls.

Variables		Cases n=86	Controls n=80	Difference	(95% CI)	p value
Weight (kg)§	Average ± SD Shapiro Wilk p-value	62.21 ± 14.70 0.89	70.52 ± 10.86 0.99	-8.31	(-12.20; -4.36)	<0.001^a^
BMI (kg/m 2)§	Average ± SD Shapiro-Wilk p-value	23.73 ± 4.72 0.01	27.5 ± 4.18 0.27	-3.87	(-5.24; -2.51)	< 0.001^b^
Total score EAT-10^A^	Median (IQR) Shapiro Wilk p-value	16 (10.5:21) <0.001	0 (0:0) <0.001	16	(14; 17)	< 0.001^c^

*§ normal distribution.* ^*A*^ *non-normal distribution. BMI: body mass index. Kg: kilograms. m: meters. EAT-10: Eating Assessment Tool. SD: standard deviation. QR: inter-quartile range. a Welch's T. b Student 's t. c Mann-Whitney U. CI: 95% Confidence Interval.*

**Figure 2:**
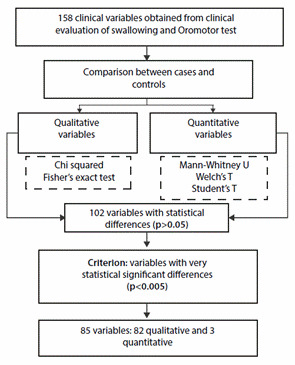
Synthesis of comparison and identification of clinical variables between cases and controls.

Figure 2 briefly shows the comparison and identification process by p value <0.005 of the clinical variables obtained in both groups.

In the 85 variables with very statistically significant differences, those with narrow confidence intervals, that had semiological meaning and accessibility by health professionals, were selected to enter them into an BLR model, verifying the VIF in each one with values between 1 and 3, and with adjustment measure by low AIC criterion. From the above, it was possible to obtain an BLR model to classify healthy patients with DON made up of nine clinical variables: two from respiratory history, two from symptoms, three from physical examination and two from findings in the gold motor test with consistency/ volume (see Table 4).

**Table 4:** Regression model with clinical variables to differentiate healthy patients from patients with neurogenic oropharyngeal dysphagia due to neurological and neuromuscular causes.

Coefficients Model - Patient/Healthy Category			
Predictor	OR (95% CI)	p value	VIF
Respiratory comorbidity	19.19 (1.54 - 238.08)	0.021	1.22
Intubation longer than one week	38.48 (4.41 - 335.13)	<0.001	1.20
Symptom of cough before swallowing	19.75 (1.52 - 256.45)	0.023	1.15
Current loss of appetite	13.69 (2.36 - 79.22)	0.003	1.14
body mass index	0.81 (0.71 - 0.92)	0.002	1.25
Alteration in having lips on the sides	33.54 (5.97 - 188.18)	< 0.001	1.36
Presence of lingual fasciculations	67.43 (5.87 - 774.81)	< 0.001	1.12
Multiple swallows with biscuit	14.37 (3.88 - 53.22)	< 0.001	1.34
Yogurt cough	62.72 (3.94 - 996.44)	0.003	1.22
Model fit measures			
Model	AIC	R^2^ N	
Clinical variables	102	0.788	

*OR: odds ratio. 95% CI: 95% confidence intervals. VIF: variance inflation factor. AIC: Akaike information criterion. R 2 N : Nagelkerke R squared .*

## Discussion

From an extensive set of clinical variables obtained in both cases and controls, it was possible to identify in a first step those that were individually different, and then together, identify those that could largely explain the phenomenon called NOD. In this way, an original and pioneering explanatory model was obtained (first consensus of variables that could feed a clinical algorithm) powered by nine variables with the capacity to explain 78.88% of the phenomenon, in this case, explaining when one is a patient with NOD from neurological and neuromuscular causes versus being healthy without dysphagia.

The model variables were obtained from the CES and performance of the motor gold test with consistency/volume. Clinical evaluation activities usually carried out by speech therapy, respiratory therapy, nursing and medical professionals, trained in swallowing and dysphagia, which can allow the potential use of models based on these characteristics to classify patients with OD and NOD in the healthcare setting.

The exploration and obtaining of models, both explanatory and predictive, on the topic of dysphagia has been carried out in the context of patients with head and neck cancer, stroke and prediction of dysphagia and aspiration, and not so much in the classification or identification of subtypes. of dysphagia (E.g. NOD). The published models use clinical variables in combination with other treatment variables, laboratory or functionality scales. One of them is the predictive model for acute dysphagia after radiotherapy for head and neck cancer, which uses clinical variables (such as age, sex and location of the tumor), treatment and dosimetry parameters plus genetic polymorphisms, in which it is reported an area under the ROC curve of 0.71^22^.

The predictive model for the presence or absence of dysphagia six months after a stroke uses clinical findings of initial VFSS such as lip closure, bolus formation, chewing, apraxia, tongue-palate contact, oral transit time, residue in vallecula or piriform sinus, laryngeal elevation and aspiration, achieving a sensitivity of 0.91 and specificity of 0.92^23^.

Another is the predictive model of aspiration in patients with OD, which uses data obtained from oral intake status, functional health status, and health-related quality of life, which obtained an AUC of 0.92^24^.

Models based on logistic regression and multilinear regression to identify risk and protection of post stroke dysphagia indicate that the type of stroke, mini-mental test score, sialorrhea severity scale, hemoglobin level and obstructive sleep apnea have a significant predictive value for the outcome^25^.

The explanatory model obtained here shows that two antecedents related to the respiratory system that are usually obtained by anamnesis (presence of COPD-type respiratory comorbidity, obstructive sleep apnea, asthma or rhinitis; and intubation longer than a week), two symptoms related to dysphagia (such as coughing before swallowing and current loss of appetite), three signs derived from the physical examination focused on swallowing (changes in body mass index especially in the underweight and normal weight range, alteration in orofacial praxis of bringing lips together the sides after verbal command or by imitation, and presence of lingual fasciculations) and two findings (which are also signs) when performing the oral motor test with consistencies/volume orally (such as the need for multiple swallows when trying to swallow hard consistency and dry like a cookie, and cough when taking on a thick consistency like yogurt), when present together, they explain 78.88% of the phenomenon of being a patient with NOD due to neurological and neuromuscular causes.

Although there are no published models focused on the joint behavior of clinical variables derived from the CES to identify and characterize the presence of NOD of neurological and neuromuscular causes (as is the explanatory model obtained in this study), there are reports on individual clinical variables and its relationship with dysphagia in general, OD and the clinical subtype of NOD.

In the context of patients with OD, repeated chest infections, bronchitis or pneumonia are considered indirect symptoms of dysphagia^11^, but repeated respiratory infections, chronic respiratory disease, pneumonia, bronchial spasms, airway obstruction, among other respiratory situations, nutritional and gastrointestinal are secondary complications, which occur more frequently in patients with NOD^28^. This is why respiratory history, including intubation for more than a week (as a possible indicator of severe respiratory disease) entered the model.

A cardinal symptom of dysphagia in general is the feeling of stickiness or difficulty in the passage of food through the digestive and upper respiratory system^4^^, ^^5^,which is related to the symptoms of cough when swallowing and hyporexia; but when analyzed together with background variables and signs on physical examination, they manage to explain the presence of NOD due to neurological and neuromuscular causes.

Clinically, spilling food through the lips, ptyalism, nasal regurgitation, cough, choking, food stuck in the throat, avoiding some food consistencies or changes in posture are direct symptoms of OD, and weight loss, bronchitis, pneumonia, time Prolonged eating, cough and changes in voice, articulation, speech or language are indirect symptoms^11^. Nasal regurgitation of food, the need for multiple swallows for a small bolus, and a history of repetitive respiratory infections point to NOD^29^. Therefore, the model obtained includes the overall behavior of direct and indirect symptoms of OD.

Deficiencies in voluntary facial movement in patients with OD may indicate the presence of a progressive neurological disease^11^. Orofacial apraxia’s represent losses of voluntary control in facial, lingual, pharyngeal or chewing in the presence of preserved muscular, reflex, spontaneous and automatic function^30^. In the search carried out, only one study was found in 60 patients with a first ischemic stroke with signs of dysphagia, in which orofacial apraxia was related to the laterality of the lesion^31^.

In the physical examination, some signs were found with similar frequencies between healthy people and patients with NOD, therefore, the finding of a sign during the physical examination in healthy people is not equivalent to the presence of dysphagia, and in patients, the presence of an isolated sign is not equivalent to the presence of dysphagia. It has as much semiological meaning as the presence of a set of them (as can be seen in the model obtained). In the case of patients with OD, whether due to functional or other causes, it is indicated that the physical examination performed in the oropharynx is usually normal^7^. An aspect that contrasts with the varied spectrum of signs present in the physical examination and gold motor test visualized and reported in the cases of this study.

Statistically significant differences were found in weight and body mass index (BMI) between healthy people and patients with NOD. Keeping in mind that these two variables are not only affected by the direct effect of dysphagia, because they are multifactorial conditions. It is reported that subjective weight loss is considered a non-specific symptom, but it supports the presence of OD^7^^, ^^32^. Weight loss secondary to reduced intake is a clinical feature suggestive of dysphagia^33^.

Oral non-tolerance to clear liquids, moist, dry or hard solids, and mixed consistencies, and especially the difficulty in swallowing cookies and yogurt observed in the cases of the study, are symptoms and signs that allow us to identify not only patients with dysphagia but specifically with NOD. Difficulty swallowing especially solids (30) and avoiding certain food textures is reported as an important symptom of OD^11^^, ^^34^.

Nearly two-thirds of the case group had the most frequently reported individual etiologies of NOD: PD, ALS, multiple sclerosis and stroke^8^^, ^^35^. NOD is the final and common consequence of several individual etiologies that end up affecting the physiology of swallowing^11^, therefore, no comparison or division was made between patients with neurological versus neuromuscular causes.

Pathophysiologically, it is reported that patients with NOD experience three basic phenomena typical of the alteration: prolonged swallowing response, delayed laryngeal closure and weak bolus propulsion with risk of aspiration^36^. Which causes several symptoms and signs that, when identified together (in this case through an BLR model), can help various health professionals (medical, nursing and therapeutic staff) to identify and classify said patients to classify them. dysphagia and initiate more specific management and rehabilitation processes.

Strengths of the study are the process of simultaneous recruitment and selection of healthy people without dysphagia and patients with NOD (achieving groups similar in age and sex, but comparable in terms of their swallowing situation), and the application in both groups of the same evaluation protocol that It included a large semiological spectrum focused on swallowing and neurological aspects. Selection and information bias was controlled in both groups, by verifying several eligibility and evaluation criteria by different professionals (both specialists) but trained in swallowing and dysphagia, who applied the same instruments, CES protocol and gold motor test.

The explanatory model obtained may be affected by the degree of swallowing, neurological and neuromuscular affection (severity) of the selected patients, because probably due to the eligibility criteria applied, they are the sickest or most symptomatic patients with NOD. It is suggested to continue with studies that include patients with OD and mild NOD, to see if the variables obtained or others that did not enter the model together manage to explain the phenomenon and classify in a similar or superior way to the results obtained, more studies of predictive models.

## Conclusion

NOD is part of the anatomical and clinical spectrum of dysphagia, and as a clinical entity it generates a series of characteristics that can be integrated into explanatory models using BLR.

Several characteristics obtained from non-invasive evaluation methodologies, focused on the clinical evaluation of swallowing and Oromotor tests, can be integrated into flowcharts or algorithms of sets of variables, potentially useful in the healthcare environment, including low-resource areas or levels of care. basic and intermediate in health where there is no access to instrumental tests.

The explanatory model obtained, fed by nine clinical variables, makes it possible to expand the evaluation spectrum carried out in patients with suspected NOD, to classify healthy people from patients with NOD, characterize the etiology (neurological and neuromuscular causes) and propose specific management. from its recognition, which is potentially achieved through the joint integration of certain characteristics obtained individually to the physical examination.
